# Treatment Outcomes in a Decentralized Antiretroviral Therapy Program: A Comparison of Two Levels of Care in North Central Nigeria

**DOI:** 10.1155/2014/560623

**Published:** 2014-06-17

**Authors:** Prosper Okonkwo, Atiene S. Sagay, Patricia A. Agaba, Stephen Yohanna, Oche O. Agbaji, Godwin E. Imade, Bolanle Banigbe, Juliet Adeola, Tinuade A. Oyebode, John A. Idoko, Phyllis J. Kanki

**Affiliations:** ^1^AIDS Prevention Initiative Nigeria, Abuja, Nigeria; ^2^Department of Obstetrics & Gynaecology, University of Jos/Jos University Teaching Hospital, Jos 930001, Nigeria; ^3^AIDS Prevention Initiative Nigeria, Jos University Teaching Hospital, Jos 930001, Nigeria; ^4^Department of Family Medicine, University of Jos/Jos University Teaching Hospital, Jos 930001, Nigeria; ^5^Department of Medicine, University of Jos/Jos University Teaching Hospital, Jos 930001, Nigeria; ^6^National Agency for the Control of AIDS, Abuja, Nigeria; ^7^Infectious Diseases & Immunology, Harvard School of Public Health, Boston, MA, USA

## Abstract

*Background.* Decentralization of antiretroviral therapy (ART) services is a key strategy to achieving universal access to treatment for people living with HIV/AIDS. Our objective was to assess clinical and laboratory outcomes within a decentralized program in Nigeria. *Methods.* Using a tiered hub-and-spoke model to decentralize services, a tertiary hospital scaled down services to 13 secondary-level hospitals using national and program guidelines. We obtained sociodemographic, clinical, and immunovirologic data on previously antiretroviral drug naïve patients aged ≥15 years that received HAART for at least 6 months and compared treatment outcomes between the prime and satellite sites. *Results.* Out of 7,747 patients, 3729 (48.1%) were enrolled at the satellites while on HAART, prime site patients achieved better immune reconstitution based on CD4+ cell counts at 12 (*P* < 0.001) and 24 weeks (*P* < 0.001) with similar responses at 48 weeks (*P* = 0.11) and higher rates of viral suppression (<400 c/mL) at 12 (*P* < 0.001) and 48 weeks (*P* = 0.03), but similar responses at 24 weeks (*P* = 0.21). Mortality was 2.3% versus 5.0% (*P* < 0.001) at prime and satellite sites, while transfer rate was 8.7% versus 5.5% (*P* = 0.001) at prime and satellites. *Conclusion.* ART decentralization is feasible in resource-limited settings, but efforts have to be intensified to maintain good quality of care.

## 1. Introduction

Nigeria bears the second largest burden of HIV infection in Africa, second only to South Africa. Of the estimated 1.4 million HIV-infected persons needing antiretroviral therapy (ART), only one-third of them were accessing treatment at the end of 2012 [1]. Universal access to ART remains a challenge in most of sub-Saharan Africa (SSA). The United Nations Millennium Development Goals (MDGs) were enacted in the year 2000 and MDG 6 advocated for universal access to ART by 2010. However, at the end of the decade, only 6.6 million (47%) of the estimated 14.2 million people eligible for treatment in low- and middle-income countries (LMIC) were accessing ART. Large-scale, vertical treatment programs in urban areas have largely been responsible for the rapid expansion of ART services in Africa, with many rural communities still lacking access to HIV and AIDS services [2–4].

Published reports indicate that ART can be delivered effectively in Africa, with individual biological and immunological responses to ART equivalent to those in high-resource settings [5–9]. National programs [10–13] have reported large-scale data of HIV treatment in both urban and rural populations [14–17]; however, delivery of HIV treatment in some settings presents unique challenges and current ART delivery models may significantly limit the accessibility of ART. To have the greatest impact on public health, HIV treatment programs will have to be decentralized and integrated into the existing health care system. Decentralization therefore is a key strategy towards achieving the MDG goal of universal access to ART services. Preliminary evidence from such rural programs has demonstrated that ART provision in rural communities is feasible, given the appropriate resources and infrastructure.

Concern has been raised that the rapid expansion of HIV services will reduce the quality of care for individuals within the programs as capacity and resources are stretched [16]. Characteristics of individuals accessing treatment may change over time, and this may affect overall outcomes; therefore monitoring treatment outcomes is essential to identify constraints or deficiencies in program performance. Guidelines for the decentralization of ART services were recently released in Nigeria and as such, there is limited data assessing outcomes at the level of program implementation. Our objectives were to explore baseline characteristics of patients enrolled in a decentralized ART program and to compare clinical and laboratory treatment outcomes between patients within the first year of highly active antiretroviral therapy (HAART).

## 2. Materials and Methods

### 2.1. Patients

The AIDS Prevention Initiative in Nigeria (APIN) and Harvard School of Public Health HIV program, supported by a grant from the United States President's Emergency Plan for AIDS Relief (PEPFAR), has supported the provision of treatment and care services to HIV-infected patients at the Jos University Teaching Hospital (JUTH), North Central Nigeria, since 2004. All patients enrolled in the JUTH PEPFAR supported program provided written consent for care; data for those that also consented for use of their information in future analyses were evaluated. The treatment protocol and written consent were approved by the institutional review boards (IRBs) at JUTH and the Harvard School of Public Health. Approval for secondary use of treatment data for this study was also obtained from the IRB at the Harvard School of Public Health.

This retrospective cohort analysis was performed using routine treatment data from patients enrolled at a tertiary hospital: Jos University Teaching Hospital (JUTH), (prime site or “hub”) and 13 secondary-level health centers (satellite sites or “spoke”) between June 2007 and May 2011. ART eligibility was based on the Nigerian National Adult ART Guidelines [16], with ART recommended for all individuals with CD4 counts <200 cells/mm^3^ and for those individuals with WHO clinical stage 3 or 4 and CD4 counts <350 cells/mm^3^. Recommended first-line ART included a nonnucleoside reverse transcriptase inhibitor (NNRTI) plus 2 nucleoside reverse transcriptase inhibitors. Once patients were assessed for eligibility, they were asked to return for both group and individual counseling and education sessions to prepare them for ART. During the first year of HAART, all patients had baseline evaluation. This was repeated at 3 and 6 months. Those with detectable viral loads at 6 months had their next evaluation at 9 months. However, those with undetectable viral loads at 6 months had their next evaluation at 12 months, provided that there were no adverse events to warrant more frequent monitoring. Laboratory monitoring for follow-up included hematological indices, biochemical parameters including liver enzymes, serum creatinine, fasting blood sugar, and total cholesterol, and CD4 count and viral load monitoring.

The study cohort consisted of ART-naive patients ≥15 years of age for whom at least 6 months of treatment follow-up were available.

### 2.2. Laboratory Analysis

CD4 count measurement was performed using laser-based flow-cytometric enumeration technique (Cyflow, Partec GmbH, Munster, Germany). Plasma HIV-1 RNA polymerase chain reaction determination was performed using the Roche Amplicor Monitor assay, version 1.5 (Roche Diagnostics, NJ, USA). All laboratories participate in regular external quality-control programs for HIV infection diagnosis, CD4+ cell count enumeration, and plasma VL estimation. At the prime site, HBsAg was determined using enzyme immunoassay (EIA) (Monolisa HBsAg Ultra3; Bio-Rad) and HCV antibody was tested using third generation EIA (DIA.PRO Diagnostic, Bioprobes srl, Milan, Italy). At the satellites, hepatitis status was determined using rapid test kits (Abon Biopharm Co., Ltd., Hangzhou, China).

### 2.3. Data Collection

Patient data were collected on standardized clinic forms and entered into a customized electronic record database (FileMaker Pro) by trained data staff at each site. Baseline evaluations included medical history, physical examination, WHO clinical staging, complete blood counts, CD4 count, and plasma VL. These same clinical and laboratory evaluations were performed 3 and 6 months after ART initiation and then approximately every 6 months thereafter unless symptoms required more frequent monitoring.

### 2.4. Description of Decentralization Model

**Figure 1:**
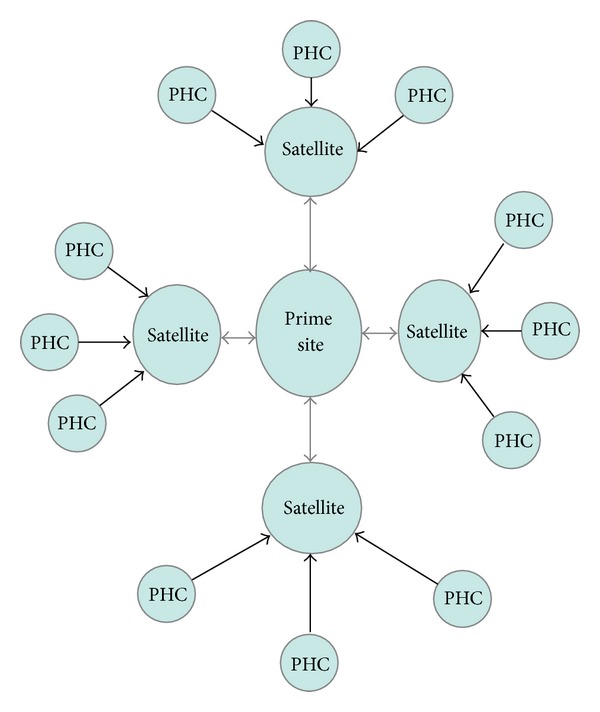
Hub-and-spoke model of ART decentralization in Jos, Nigeria (PHC: primary health centre; ↔ two-way flow of referrals; and → one-way flow of referrals).

From June 2007, the Harvard PEPFAR/APIN Plus program at JUTH commenced statewide provision of ART services in Plateau State. This was accomplished using the “hub-and-spoke” model (Figure 1). The hub (prime site) was the urban-located JUTH from where services were decentralized to the 13 satellite sites within Plateau State. The satellite sites were secondary-level hospitals located at semiurban and rural communities within different local government areas in the state. The satellite clinics were a mix of government, faith-based, and for-profit centres. They all were secondary-level facilities and offered general medical services including surgical, obstetrics, and pediatric care. They had different resource bases, but for the implementation of comprehensive HIV/AIDS services, their systems received the same level of support and equipment.

None of the satellite sites had experience with implementation of comprehensive ART services. However, all of them were providing HCT and single-dose nevirapine based PMTCT services prior to the scale-out process. Each of the secondary-level facility was in turn linked to at least three primary health centers (PHCs).

The first step began in 2006 with site assessment and selection. Sites were chosen based on the following criteria: (i) availability of medical doctors/nurses/laboratory scientists/pharmacists and other support staff; most cadres of health care personnel were present at the satellite sites; however, they were present in limited numbers compared to the tertiary site where the absolute numbers of staff were higher; (ii) available HIV testing and counseling (HTC) services; (iii) the presence of adequate counseling and consulting rooms; (iv) capability for safe and secure storage of drugs; and (v) number of patients in need of treatment. Facilities that had adequate patient load but failed to meet the minimum criteria received health systems strengthening and logistical support to operate comprehensive HIV services. The next step involved capacity building and training of health workers using standardized national training tools. In addition to centrally organized trainings, each satellite site received weekly mentorship visits from the satellite team. The team comprised of specialist physicians, an obstetrician, nurse counselors, data personnel, laboratory scientist, and pharmacist. The team visited the sites on their clinic days and provided hands-on training. The third step involved the roll-out and implementation of comprehensive ART services through integration of ART follow-up visits with primary outpatient care services at the satellites. Patients were decentralized for ART follow-up from the prime site to the satellites if they met several criteria: (i) adults or older children on first-line ART (or alternative first-line) for more than 6 months and stable; (ii) no evidence of active opportunistic infection or drug intolerance; (iii) ART provider's confidence in patient adherence; at enrollment, all patients were given general HIV/ART counseling on one-on-one basis and had at least two contacts with the counselors before commencement of HAART; in addition, adherence was assessed at each clinic visit using patient recall and our electronic pharmacy database; and (iv) patient living closer to satellite than to prime site.

The details of the various roles of health care staff at both levels of care are shown in Table 1.

### 2.5. Statistical Analysis

Differences between groups were compared using the Chi-square test for categorical variables and *t*-tests for continuous variables. We compared immunovirologic outcomes between the prime and satellite sites at 24 and 48 weeks. We also compared change in body weight, hemoglobin levels, and time to HAART initiation (defined as the period between enrollment for care and the pick-up of the first ART prescription) between the sites. Cox proportional hazard model was used to evaluate the relationship between decentralization and the risk of death. In addition, we assessed the risk of virologic failure (VF) (viral load >1000 copies/mL as per protocol) at 12 months of follow-up using a logistic regression model. The 48-week end point viral load data was available for 648 patients (403 and 281 at the prime and satellite sites, resp.) and these were the number of patients analyzed for the VF outcome. Adjustments in multivariate analysis were made for sex, age, clinical stage at initiation, baseline anemia, elevated creatinine, CD4 count, program site, and educational status. Data analysis was performed using Epi Info version 3.5.3 (CDC, Atlanta, GA, USA) and plots created using the Microsoft Excel graph feature. *P* values <0.05 were considered significant.

## 3. Results

**Figure 2:**
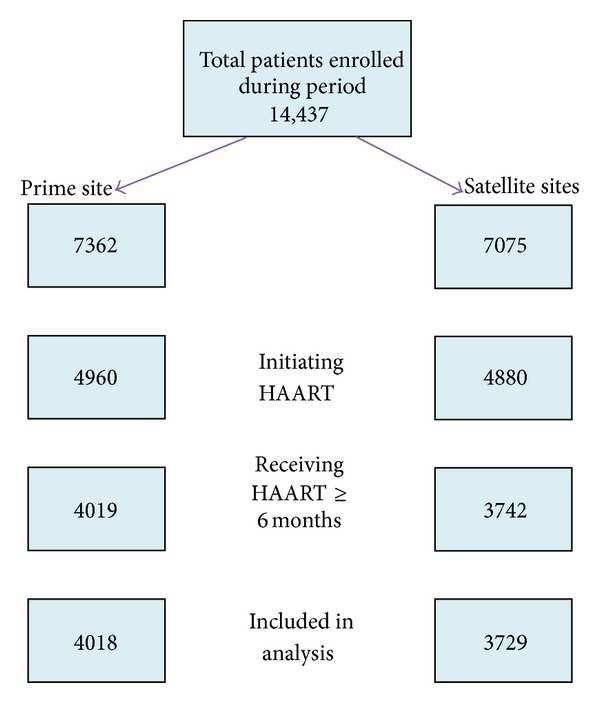
Flow chart of patients included in the study.

The enrollment of patients by site and flow from recruitment to inclusion in this study is shown in Figure 2. Overall, females (5381) made up 69.5% of all participants reported in this study and 609 (11.3%) were pregnant at the time of enrollment. The mean age was 34 ± 9 years with males enrolling for care at older age compared to females (39 ± 9 versus 32 ± 8 years, *P* < 0.001). 4712 (60.8%) were married and 1394 (17.8%) reported having no formal education. The median CD4 count at enrollment was 180 cells/mm^3^ (IQR: 4–1481) and males enrolled for care at a lower CD4 count compared to females (152 versus 195 cells/mm^3^, *P* < 0.001). A higher proportion of males (65%) than females (51.2%) (*P* < 0.001) entered care with CD4 < 200 cells/mm^3^. There were 2195 (28.3%) patients with advanced WHO disease and the prevalent TB rate for the entire cohort was 10.1%. The details of baseline comparison between patients at the satellite and prime sites are shown in Table 2.

### 3.1. Comparison of Outcomes

**Figure 3:**
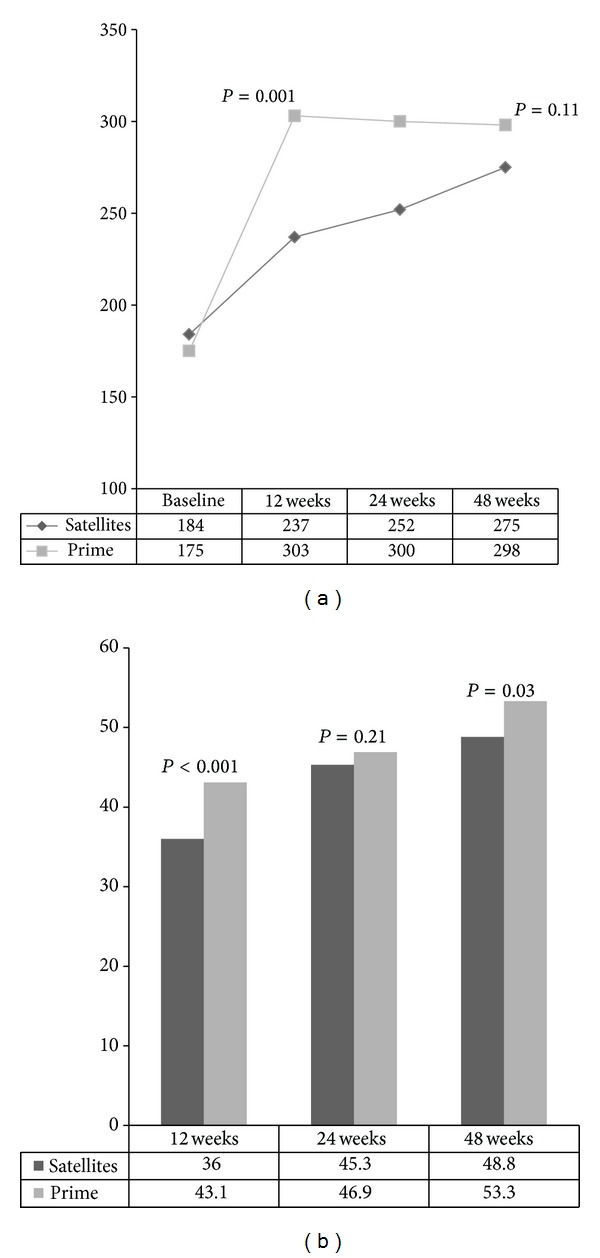
(a) Line graph showing trend in median CD4 levels (cells/mm^3^) at baseline through 48 weeks of follow-up between patients at the prime and satellite sites in Jos, Nigeria. (b) Bar chart showing proportions of patients achieving undetectable viral load (<400 copies/mL) from 12 through 48 weeks of follow-up in patients at the prime and satellite sites in Jos, Nigeria.

For immunologic outcome, we compared absolute CD4 count as well as median increase from baseline over time points (Table 3 and Figure 3(a)). In spite of presenting with significantly lower CD4 counts at baseline, patients at the prime site had significantly higher median increases at all subsequent follow-up visits and also in median absolute counts except at the 48-week visit where there was no difference between the sites in absolute CD4 count. The proportion of patients with undetectable VL levels was higher at 12 and 48 weeks among patients at the prime site, compared to those at the satellites (Figure 3(b)). Virologic suppression rates were similar at 24 weeks between the two groups.

Time in care refers to time since enrollment, which encompasses both the pre-ART and ART periods, whereas time on HAART refers only to the period spent on HAART. These two end points differed significantly between the satellites and prime site at the end of one year. Time to ART initiation was significantly longer at the satellite sites, compared with the prime site (*P* < 0.001). There was no significant difference in the weight and hemoglobin levels of the patients at the two sites at the end of the first year of treatment. The details of clinical and laboratory outcomes and follow-up data are shown in Table 3. The number of deaths reported differed significantly over the 12-month follow-up period between the prime and satellite sites. Based on our model, patients could be transferred from the tertiary site to the satellites and also from the satellites to the tertiary site. One of the objectives of the model was to move treatment services closer to the patients. Our rate of transfer captures patients who may have moved from the tertiary to the satellite sites for the tertiary site and from the satellites to the tertiary site for the satellite sites. The transfer rate was significantly higher for the tertiary site compared to the satellite sites.

When we adjusted for sociodemographic and biologic variables, the factors associated with mortality in time-to-event analysis were male sex, older age, enrollment at a satellite site, low baseline hemoglobin, and CD4 < 50 cells/mm^3^. Similarly, the factors predictive of virologic failure at 12 months were advanced WHO stage disease and CD4 count >200 cells/mm^3^ (Table 4).

## 4. Discussion

An estimated 1.5 of Nigeria's 3.5 million HIV positive persons are in need of ART; however only a third of this number is currently receiving these lifesaving medicines [1]. In order to meet her treatment targets, there is need to explore different models of decentralization of HIV services. Decentralization of ART services is a key strategy for the expansion of treatment access to people living with HIV (PLHIV). Since 2007, our program utilized the hub-and-spoke model to scale out ART from a congested tertiary-level hospital to 13 secondary-level health facilities. Scaling down HIV treatment to lower levels of care appeared to be both feasible and effective. However, in our study, poorer outcomes were found at lower level clinics compared to the primary hub hospital. Therefore, further measures to optimize the provision of care will be needed to fully sustain the long-term benefits of decentralization.

We found the risk of death to be higher for patients enrolled at the satellites compared to the prime site. This finding agrees with a study in Malawi [18], which reported higher mortality among patients seen at health centers compared to hospital setting. However, another study in Malawi [12] reported lower death rates at health centers in a decentralized ART program. Whereas the ART program in Malawi used task shifting with downreferral of stable, less ill patients to health centers from hospital setting, our hub-and-spoke model entailed the secondary-level facilities enrolling new patients irrespective of clinical status and encouraged the referral of sick/unstable patients to the tertiary hospital after an initial triage period. Our overall mortality for both levels of care was lower compared to similar cohorts in SSA [19], but the risk factors for death were similar to other cohorts from the countries most affected by HIV; these are male sex, anemia, and low baseline CD4 count [18, 20–22]. A possible explanation for our lower rates may be our inability to account for all deaths, as our database was not directly linked to any public health records/registry and mortality reporting was passive.

One key measure of HIV treatment success is the ability to sufficiently reconstitute the immune system to levels where opportunistic infections (OIs) become less common and cease to threaten survival in PLHIV. Achieving and maintaining adequate level of CD4 counts is important as many OIs are prevented when CD4 counts rise above 200 cells/mm^3^. Although our patients presented with very low baseline CD4 counts at both levels of care, the two groups achieved impressive immunologic response to ART in the first year with patients at the prime site achieving significantly higher increases. Studies reporting immunologic response to ART in Africa [23–25] have obtained similar margins of CD4 increase among patients on ART, with slightly lower increases among older age groups. The use of prophylactic agents such as cotrimoxazole among other initiatives will help to further improve immune function and sustain the benefits of ART as programs scale out to rural areas with high burden of OIs.

Routine virologic monitoring, though uncommon in HIV treatment programs in resource-limited settings, remains the gold standard for early detection of treatment failure. Recent endorsement by WHO of virologic monitoring for patients on HAART gives the best picture of treatment response, and the ability to maximally suppress viral loads to undetectable levels is a key outcome in ART programs. We found more than half of our patients achieving undetectable viral loads at one year of treatment. Studies [26–29] have reported varying rates of virologic response to treatment in settings similar to ours with majority reporting higher rates of viral suppression than we observed in our study. A large study from South Africa, which compared treatment outcomes between three levels of care, reported higher likelihood of virologic suppression as well as other outcomes at primary health centres, compared to regional and district level hospitals [30]. Even though our study did not include any primary health centre, one reason that may be responsible for the poorer outcomes we reported at the satellites could be the constraint of limited staffing which was a recurring issue at all of our satellite sites. Although we did not assess medication adherence in this study, poor or suboptimal adherence has been reported to be largely responsible for treatment failure in ART programs across SSA. For treatment programs to ensure sustained adherence in the long term, medication adherence support initiatives need to be an integral part of procedures irrespective of the model of service delivery.

We used time to ART initiation as one of our outcome measures in this study and found that patients at the satellite sites had longer waiting time to commencement of ART compared to patients at the prime site. This may be explained by the fact that the prime site was better resourced and could decide ART eligibility at an earlier rate. Studies reporting delays in ART initiation have cited various factors being responsible for such delays [31–34]. Timely initiation of ART in patients enrolling for care has implications for early mortality, loss to follow-up, and other outcomes during subsequent follow-ups. Delays in initiation of ART could be due to health system, or patient-related factors, including pre-ART attrition and system-mandated delays to allow for appropriate pretreatment counseling [35, 36]. These delays were associated with excess rates of preventable morbidity and mortality, especially in ART-eligible patients with advanced HIV disease who are already at elevated risk of death. The use of point-of-care testing and simplified guidelines, protocols, and regimens, coupled with efficient triaging of new patients, will serve to minimize some of these unnecessary delays in the context of decentralization.

The treatment model described here, a decentralized program with treatment delivered through the hub-and-spoke model, has allowed rapid scale-up, with an additional 7075 adult patients enrolled for care with 4880 initiating ART in a 4-year period. The rapid increase of ART initiation in the satellite sites is encouraging and suggests that the availability of care at the secondary level reduces the common barriers (travel time and cost) to ART uptake. Other aspects of HIV care, such as pre-ART monitoring and long-term adherence support, will be required to maintain delivery of high quality care as we scale comprehensive HIV services down to secondary and primary levels of care.

One of the strengths of this study is that our data originates from operational real-life program information collected as part of routine monitoring and evaluation from a public donor-supported HIV program in a high burden country and may therefore reflect the challenges faced with implementation of public sector programs in many resource-constrained settings. Secondly, we have an electronic medical record system that includes clinical laboratory and pharmacy data, which improved access to longitudinal data for the over 7000 patients included in this analysis. Thirdly, outcomes such as CD4 counts and viral loads required optimal adherence to the APIN ART monitoring protocol. One of our concerns was regarding the accuracy and consistency associated with use of operational data. Differential censoring due to missing data between different levels of care could not be completely discounted. Our mortality estimates may be underestimates because we did not have access to any public registries. However, our program has a tracking unit that routinely generates a list of patients lost to follow-up and this enabled us to document the exact disposition of some patients who may have died outside of the health care setting. Finally, our study is limited by the short follow-up of 12 months and further studies will be required to evaluate differences in health care provision and outcomes beyond the first year of ART, particularly retention and subsequent viral suppression rates.

In conclusion, we have shown that rapid scale-up of ART delivery through a decentralized secondary-level care program is feasible in our resource-limited setting and produces outcomes comparable to those reported in other SSA settings. The high rates of detectable viral load in patients on ART at our satellite sites highlight the challenges of program implementation at less well-resourced health centers and efforts have been made to intensify support for medication adherence and to maintain quality of care by health systems strengthening as we scale out to secondary and primary facilities.

## Figures and Tables

**Table 1:** Profile and role of health workers at the prime and satellite sites in the decentralized ART program in Jos, Nigeria.

Category	Prime site	Satellite sites
Physicians	Specialist physicians running daily adult, pediatric, and PMTCT services with daily mentoring of junior staff. Concurrent activities include baseline assessment, ART initiation, review of difficult cases, and switch to second-line ART	General duty doctors providing integrated services 1-2 days a week. Weekly mentoring visits by the satellite team with provision of technical assistance and logistical support

Pharmacists	Several pharmacists with dedicated adult and pediatric tracks. Assisted by pharmacy assistants	One pharmacist for each performing combined adult and pediatric dispensing on clinic days. Some are assisted by pharmacy technicians/assistants

Nurses	Trained nurse/midwives with specialized training in ART triage, reproductive health, and adult, pediatric, and PMTCT services	Trained nurse/midwives providing integrated HIV services at sites. Some sites have auxiliary nurses

Patient care attendants	Trained adherence and medical health records personnel dedicated to adult/pediatric/PMTCT clinics. Nutrition counselors as well as clinical psychologists providing general HIV/AIDS prevention education and nutritional counseling	Nurses trained to provide general HIV/AIDS and nutritional counseling

Expert patients	Trained expert PLWA provide adherence counseling, vital sign measurement, anthropometry, and defaulter/loss to follow up tracking	Available at some sites

Home-based care volunteers/CBO	HBC volunteers provide peer support and promote community care and services among PLWA. CBOs not utilized	CBOs provide community-based OVC services

**Table 2:** Comparison of baseline characteristics between patients enrolled at the prime and satellite sites in the decentralized ART program in Jos, Nigeria.

Characteristic	Satellite (*n* = 3729)	Prime (*n* = 4018)	*P* value
Proportion enrolled	48.1	51.9	
Sex			
Female	2700 (72.4)	2681 (66.7)	<0.001
Male	1029 (27.6)	1337 (33.3)	
Missing	0 (0.0)	0 (0.0)	
Age, years			
Median (IQR)	32 (15–80)	33 (15–81)	0.001
Missing	3	0	
Marital status			
Married	2390 (64.1)	2322 (57.8)	<0.001
Single	531 (14.2)	898 (22.3)	
Other	793 (21.3)	798 (19.8)	
Missing	14 (0.4)	0 (0.0)	
Educational attainment			
No formal education	719 (19.3)	665 (16.5)	0.004
Primary	1148 (30.7)	862 (21.5)	
Secondary	1107 (29.7)	1260 (31.4)	
Tertiary	693 (18.6)	1221 (30.4)	
Missing	62 (1.7)	10 (0.2)	
Number unemployed	758 (21.3)	792 (19.9)	0.49
Missing	125 (3.3)	0 (0.0)	
WHO staging			
1	1718 (46.1)	1429 (35.6)	<0.001
2	948 (25.4)	752 (18.7)	
3	585 (15.7)	1257 (31.3)	
4	179 (4.8)	174 (4.3)	
Missing	299 (8.0)	406 (10.1)	
Number with tuberculosis*	324 (11.1)	70 (7.5)	<0.001
Missing	254 (6.8)	953 (23.7)	
High creatinine (>120 *μ*mol/L)	205 (5.5)	276 (6.8)	
Missing	17 (0.5)	48 (1.1)	
Severe anaemia (Hb < 8 g/dL)	209 (5.6)	155 (3.9)	0.0003
Missing	15 (0.4)	8 (0.1)	
Median CD4 (cells/mm^3^), IQR	184 (4–1481)	175 (5–1243)	0.0001
Missing	3 (0.0)	1 (0.0)	
Advanced HIV disease**	2008 (53.8)	2284 (56.8)	<0.008
Median viral load (copies/mL)	37 357	43 456	0.04
Missing	389 (10.4)	12 (0.2)	
Hepatitis B positive	454 (13.1)	776 (22.7)	<0.001
Missing	227 (6.0)	605 (15.0)	
Hepatitis C positive	308 (8.9)	522 (14.7)	<0.001
Missing	281 (7.5)	455 (11.3)	
Number of pregnant females	360 (13.5)	249 (9.8)	<0.001
Missing	25 (0.6)	131 (3.2)	

*Both pulmonary and extrapulmonary; **CD4 < 200 cells/mm^3^.

**Table 3:** Outcomes through 12 months of follow-up among patients initiating antiretroviral therapy in a decentralized HIV treatment program (2007–2011) in Jos, Nigeria.

Variable	Satellite sites	Prime site	*P* value
Time to HAART initiation (days)	84	32	<0.001
Time in care (months)	23 (6–98)	34 (6–98)	<0.001
Duration on HAART (months)	17 (0.4–59)	23 (0.3–59)	<0.001
Median CD4 increase (cells/mm^3^)			
12 weeks	53	128	<0.001
24 weeks	68	125	<0.001
48 weeks	91	123	0.03
Change in body weight (kg), mean (SD)			
Baseline	57.1 (11.4)	58.6 (11.9)	<0.001
12 weeks	57.0 (10.7)	59.1 (11.8)	<0.001
24 weeks	57.1 (10.9)	58.5 (11.6)	0.003
48 weeks	57.5 (11.7)	58.2 (11.5)	0.11
Change in haemoglobin (g/dL)			
12 weeks	11	11	0.001
24 weeks	11.5	11	0.68
48 weeks	11.8	12	0.39
Transfers (%)	205 (5.5)	349 (8.7)	<0.001
Deaths (%)	201 (5.0)	85 (2.3)	<0.001

**Table 4:** Risk factors for death within 12 months and virologic failure in patients initiating ART in a decentralized HIV treatment program (2007–2011) in Jos, Nigeria.

Variable	Death			Virologic failure		
	AHR	95% CI	*P* value	AHR	95% CI	*P* value
Sex						
Female	1			1		
Male	1.60	1.06–2.42	0.02	0.88	0.66–1.18	0.40
Age (years)						
<35	1			1		
35–49	1.66	1.07–2.56	0.02	1.22	0.66–2.26	0.52
≥50	1.21	0.63–2.30	0.55	1.11	0.52–2.35	0.77
Baseline CD4 (cells/mm^3^)						
≥200	1			1.30	1.00–1.69	0.04
51–199	0.87	0.56–1.34	0.53	1.50	0.34–6.54	0.58
≤50	2.3	1.42–3.70	0.0006	5.13	1.93–13.65	0.001
Program level						
Prime site	1			1		
Satellite sites	5.05	3.31–7.70	<0.001	0.98	0.77–1.26	0.92
Haemoglobin (g/dL)						
≥8	1			1		
<8	2.86	1.30–6.29	0.008	1.16	0.67–2.01	0.57
WHO stage						
1 and 2	1			1		
3 and 4	0.99	0.66–1.49	0.98	1.50	1.22–2.06	0.0005
Creatinine (*μ*mol/L)						
≤120	1			1		
>120	1.09	0.59–2.01	0.77	1.05	0.63–1.76	0.83
Formal education						
Any	1			1		
None	0.85	0.51–1.43	0.57	0.85	0.60–1.20	0.36
